# Activation Ratio Correlates with IQ in Female Carriers of the *FMR1* Premutation

**DOI:** 10.3390/cells12131711

**Published:** 2023-06-24

**Authors:** Dragana Protic, Roberta Polli, Ye Hyun Hwang, Guadalupe Mendoza, Randi Hagerman, Blythe Durbin-Johnson, Bruce E. Hayward, Karen Usdin, Alessandra Murgia, Flora Tassone

**Affiliations:** 1 Department of Pharmacology, Clinical Pharmacology and Toxicology, Faculty of Medicine, University of Belgrade, 11000 Belgrade, Serbia; dragana.protic@med.bg.ac.rs; 2 Laboratory of Molecular Genetics of Neurodevelopment, Department of Woman and Child Health, University of Padova, 35128 Padova, Italy; roberta.polli@unipd.it (R.P.); alessandra.murgia@unipd.it (A.M.); 3 Fondazione Istituto di Ricerca Pediatrica, Città della Speranza, 35128 Padova, Italy; 4 Department of Biochemistry and Molecular Medicine, School of Medicine, University of California Davis, Sacramento, CA 95817, USA; yehhwang@ucdavis.edu (Y.H.H.); gumendoza@ucdavis.edu (G.M.); 5 Medical Investigation of Neurodevelopmental Disorders (MIND) Institute UCDH, University of California Davis, Sacramento, CA 95817, USA; rjhagerman@ucdavis.edu; 6 Department of Pediatrics, School of Medicine, University of California Davis, Sacramento, CA 95817, USA; 7 Department of Public Health Sciences, Division of Biostatistics, University of California, Davis, CA 95616, USA; bpdurbin@ucdavis.edu; 8 Laboratory of Cell and Molecular Biology, National Institute of Diabetes, Digestive and Kidney Diseases, National Institutes of Health, Bethesda, MD 20892, USA; bruce.hayward@nih.gov (B.E.H.); karenu@niddk.nih.gov (K.U.)

**Keywords:** *FMR1* mRNA, CGG, premutation carriers, activation ratio, IQ, depression, methylation

## Abstract

Carriers of the *FMR1* premutation (PM) allele are at risk of one or more clinical conditions referred to as FX premutation-associated conditions (FXPAC). Since the *FMR1* gene is on the X chromosome, the activation ratio (AR) may impact the risk, age of onset, progression, and severity of these conditions. The aim of this study was to evaluate the reliability of AR measured using different approaches and to investigate potential correlations with clinical outcomes. Molecular and clinical assessments were obtained for 30 PM female participants, and AR was assessed using both Southern blot analysis (AR-Sb) and methylation PCR (AR-mPCR). Higher ARs were associated with lower *FMR1* transcript levels for any given repeat length. The higher AR-Sb was significantly associated with performance, verbal, and full-scale IQ scores, confirming previous reports. However, the AR-mPCR was not significantly associated (*p* > 0.05) with these measures. Similarly, the odds of depression and the number of medical conditions were correlated with higher AR-Sb but not correlated with a higher AR-mPCR. This study suggests that AR-Sb may be a more reliable measure of the AR in female carriers of PM alleles. However, further studies are warranted in a larger sample size to fully evaluate the methylation status in these participants and how it may affect the clinical phenotype.

## 1. Introduction

The Fragile X Messenger Ribonucleoprotein 1 (*FMR1*) gene is located on chromosome Xq27.3 and encodes the *FMR1* protein, FMRP. The 5′ untranslated region of a normal allele of the *FMR1* gene contains a tandem repeat tract harboring 5–44 CGG trinucleotide repeats, while premutation (PM) alleles contain 55–200 CGG repeats. The prevalence of PM in the general population is about 1:855 in males and 1:300 in females [1]. The PM carriers can present with (i) neuropsychiatric conditions recognized as fragile X-associated neuropsychiatric disorders (FXAND) [2], (ii) conditions leading to fertility problems and early menopause in females, named fragile X-associated primary ovarian insufficiency (FXPOI) [3], and (iii) early onset of a neurodegenerative disorder named fragile X-associated tremor/ataxia syndrome (FXTAS) [4], which all fall under the umbrella of Fragile X premutation-associated conditions (FXPAC).

The *FMR1* gene in PM carriers is overactive, expressing higher levels of *FMR1* mRNA [5]. The increased RNA expression leads to RNA toxicity [6], which is associated with (i) intranuclear inclusions within neurons and astrocytes, (ii) Ca^2+^ dysregulation, (iii) mitochondrial dysfunction [4], (iv) repeat-associated non-AUG (RAN) translation with a major product protein known as FMRpolyG [4,7,8], and (v) DNA damage caused via R-loop formation [9].

As the size of the CGG repeat tract increases, the PM allele displays increasing degrees of repeat instability within tissues and across generations [10,11,12]. In particular, intergenerational CGG allelic instability is well documented for PM alleles which may further expand to a full mutation (FM; >200 CGG repeats) in the next generation [4,13]. The *FMR1* FM causes Fragile X syndrome (FXS), the most common form of inherited intellectual disability (ID) and monogenic cause of autism spectrum disorder (ASD). The FM becomes epigenetically silenced, leading to a reduction or absence of FMRP [14]. The risk of passing FM to the offspring increases with the length of the maternal PM allele. It is nearly 100% for mothers with >90 CGGs [15], but it is affected by the presence of AGG interruptions and by maternal age [16].

Furthermore, our group has demonstrated somatic instability in the blood of female carriers of PM alleles [10]. According to this study, the number of CGG repeats in the originally inherited allele and age were both positively connected with instability, while the number of AGG interrupts was negatively correlated. This study also showed that additional genetic variables, particularly genes involved in DNA repair, determine how much somatic CGG instability there is in female PM carriers [10].

The activation ratio (AR), which represents the fraction of the normal allele carried on the active X chromosome, has been shown to be a relevant clinical parameter in many diseases involving X-linked genes, including hemophilia B [17,18], Duchenne muscular dystrophy [18], myotubular myopathy [19], Fabry disease [20,21], and dyskeratosis congenita [22]. In the case of the *FMR1* gene, if skewed X chromosome inactivation (XCI) occurs, resulting in more of the normal allele on the inactive X, the lack of FMRP in FM allele carriers may result in a more severe clinical phenotype. Indeed, several studies have reported how methylation status and AR significantly impact the degree of neurocognitive and physical phenotypes in females with *FMR1* FM [23,24,25]. In theoretical terms, the AR could have an effect on the risk, severity, and age of onset of FXPAC in female PM carriers because the transcription of such alleles is linked to RNA toxicity and considering the amount of FMRP may be reduced as a consequence of the presence of numerous repeats in the 5′ UTR that affect translation [26]. Although more research is needed to determine how AR and disease pathophysiology interact in female carriers of PM alleles, this measure should be taken into account when genotype/phenotype correlations are made, as the AR contributes to the activity of the *FMR1* gene.

The Human Androgen Receptor Assay (HUMARA) is considered the gold standard for determining X inactivation status [27]. However, this assay, which is based on the methylation status of the androgen receptor (AR) gene located near the X inactivation center, does not identify whether it is the normal X or the X carrying the PM that is preferentially inactivated. Measurements of methylation status and of AR can also be accomplished using Southern blot analysis on genomic DNA digested with both a methylation-insensitive restriction enzyme and a methylation-sensitive one [28,29] or using methylation-sensitive PCR (mPCR). The latter assay compares the ratio of the PCR products corresponding to the normal allele seen with and without pre-digestion with a methylation-sensitive restriction enzyme [30]. However, both methods have disadvantages; AR-Sb analysis is a time-consuming process and requires a relatively large amount of DNA (~10 µg) from fresh blood, while PCR is susceptible to size bias, particularly in sequences that are difficult to amplify, such as long stretches of CGG repeats. However, to date, no systematic comparison has been conducted to compare the correlation of AR-mPCR and AR-Sb with clinical outcomes. Here, we report the correlations between AR and clinical outcome in 30 female carriers of a premutation allele using both the AR-mPCR and AR-Sb assays.

## 2. Materials and Methods

### 2.1. Participants

Participants were 30 female PM carriers whose PM status (CGG repeat size 55–200) was confirmed via both Sb and PCR approaches (Table 1). The females were included either as part of a dedicated research visit or following cascade testing after consultation for a child or sibling with FXS. The study and all research protocols were carried out in accordance with the Institutional Review Board (IRB) at the University of California, Davis, with written informed consent obtained from all participants in accordance with the Declaration of Helsinki.

### 2.2. Molecular Data

Molecular data were obtained from peripheral blood samples (3 mL), and the genomic DNA was isolated from 30 PM females using the Gentra Puregene Blood Kit (Qiagen, Valencia, CA, USA) and utilized for measuring the size of CGG trinucleotide repeats (expansion), a number of the AGG interruptions, and methylation status of the *FMR1* gene. The assessment of the *FMR1* CGG repeat allele size was performed using PCR and Sb analysis as previously described [10,28,31]. Capillary electrophoresis (CE) was used for the visualization and sizing of the PCR products. As reported previously, triplet-primed PCR protocol was used for the detection of the number of AGG interruptions [32,33]. Visualization and analyses of AGG interruptions were carried out using CE and Peak Scanner Software 2.0.

The X-inactivation status was determined using an assay based on a short polymorphic repeat tract present in the X-linked androgen receptor (HUMARA) gene as previously described [27]. Allele peak areas were analyzed using an ABI 3130 automated sequencer and the GeneScan software (PE Applied Biosystems). The fraction of the normal *FMR1* allele on the active X chromosome (AR) was assessed using both Sb and mPCR analysis [34,35]. AR was evaluated as an indicator of the percentage of cells bearing the normal allele on the active X-chromosome via two separate Sb runs for all samples (technical replicates), as described in Tassone et al. (1999) [34], and is given here as AR-Sb1 and AR-Sb2; it was also evaluated once using mPCR analysis, which is indicated as AR-mPCR. The matrices of measurement of instability as the differences in the number of repeats in the CE profile between the expanded allele (Peak 2) and stable allele (Peak 1) were previously described in detail by Hwang and colleagues (2022) [10].

The *FMR1* total mRNA was isolated from peripheral blood collected in PAX gene collection tubes (Qiagen, Valencia, CA, USA) and quantified using the Agilent 2100 Bioanalyzer system. *FMR1* mRNA levels were measured using qRT-PCR used Assays-On-Demand (Applied Biosystems, Foster City, CA, USA) and custom TaqMan primers and probe assays as reported in Tassone et al. (2000) [5].

### 2.3. Clinical Assessment

Clinical assessment, following under the umbrella of FXPAC, included the following: (i) presence of FXTAS, FXPOI, and FXAND; (ii) number of psychiatric conditions related to FXAND, which included depression, anxiety, ASD, and attention deficit hyperactivity disorder (ADHD), occurring at the same time; (iii) Intelligence Quotient (IQ) as measure of a cognitive assessment; (iv) presence of executive functioning deficits.

- An expert medical professional (RJH) evaluated the presence and severity of FXTAS and FXAND in patients with PM after conducting a thorough medical examination and reviewing the patients’ Magnetic Resonance Imaging (MRI) pictures. Occurrence of early menopause prior to 40 years of age, followed by medical examination, defined as FXPOI [3] was also assessed.
- The presence of psychiatric conditions related to FXAND occurring at the same time was determined using (i) the Structured Clinical Interview for DSM Disorders (SCID-5), a semi-structured interview guide for making diagnoses of anxiety, depression, and ADHD according to the diagnostic criteria published in DSM-5 and the Symptom Checklist-90-R (SCL-90 R) [36,37] and (ii) Autism Diagnostic Observation Schedule, Second Edition (ADOS-2) assessments for making diagnosis of ASD. The results were presented as the occurrences of ASD, anxiety, ADHD, and depression. The total number of these medical conditions was presented as 0, 1, 2, 3, or 4 conditions occurring in one female PM carrier at the same time [36].
- The cognitive assessment, based on standardized testing, included the Stanford Binet Intelligence Scales, Fifth Edition (SB-5) [38], and the Wechsler Adult Intelligence Scale Fourth Edition (WAIS-IV) [39]. The presented results included performance IQ, verbal IQ, and full-scale IQ (FSIQ).
- The Behavioral Dyscontrol Scale-2 (BDS-2), a nine-item assessment, was used to measure executive functioning deficits, as prediction of functioning in daily life [40,41].

### 2.4. Statistical Analysis

Statistical analyses were performed via R version 4.2.1 (23 June 2022) [42], and the Bland–Altman plots [43] were used to assess agreements between repeated AR measurements and AR measurements from different methods. For the AR measured via Southern Blot analysis, the AR-Sb2 values were used for all correlations, given that no differences were observed between Sb-1 and Sb-2. Bland–Altman plots illustrate differences between methods by showing the difference between two methods plotted against their mean. The correlation between repeated AR measurements and AR measurements from different methods was estimated and tested using the Pearson correlation. The relationship between pairs of continuous variables was analyzed using linear regression, and the relationship between continuous variables and number of AGG interruptions was analyzed using analysis of variance. Instability data were log transformed prior to analysis in order to minimize the impact of extreme values. Linear regression analyses and sensitivity analyses modeling *FMR1* mRNA via AR (AR-Sb and AR-mPCR) and CGG, using generalized additive models (GAMs), in which the relationship between *FMR1* mRNA and AR was allowed to be nonlinear, were performed.

## 3. Results

### 3.1. Study Participants

Participants in this study were 30 carrier females, and the ages of the participants in this study at the time their blood was drawn was 60.5 ± 11.7 y. (range: 40.0–84.0 y.; Median 59.0 y.). Among them, three did not have children, and 14 PM (46.7%) had a child with FXS. The average years of education in this cohort were 15.7 ± 2.6 (n = 21). Molecular measures, including CGG repeat number, number of AGG interruptions, AR, instability, and FMR1 mRNA levels are shown in Table 1.

The clinical presentation of this cohort is also summarized in Table 1. As presented, 12 participants (mean age: 65.25 ± 13.15 yo; range: 40–84 yo; median: 66.5 yo) were diagnosed with FXTAS, while 18 carriers did not have symptoms or meet the criteria of FXTAS diagnosis (mean age: 57.33 ± 9.80 yo; range: 40–80 yo, median: 57 yo). In addition, 11/24 carriers (missing data for 6 participants) were diagnosed with FXPOI (mean age: 59.27 ± 8.71 yo; range: 40–71 yo; median: 59 yo). The mean age of participants diagnosed with FXPOI, at the time when menopause occurred, was 36.0 ± 3.71 yo. In addition, anxiety and depression, as the most frequent manifestations of FXAND, were presented in 22 (mean age: 60.64 ± 10.87 yo) and 20 participants (mean age: 58.10 ± 9.91), respectively.

### 3.2. Analysis of Molecular and Clinical Data

#### 3.2.1. XCI, AR-Sb1, AR-Sb2, and AR-mPCR

**Figure 1:**
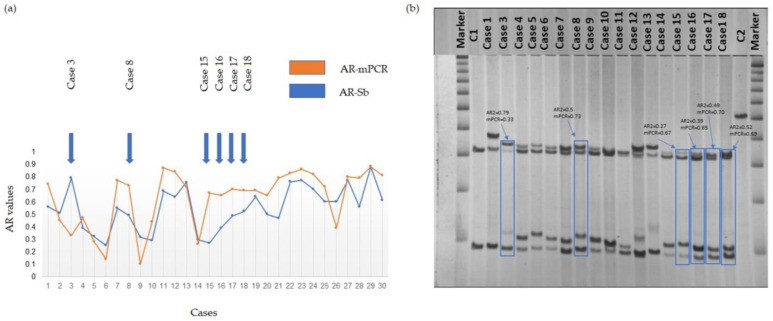
(**a**) Graphical representation of the distribution of AR values for all 30 participants obtained using the two different methods. (**b**) Southern blots (Sb) of a representative selection of a subgroup of the cohort included in this study. Cases showing a significant difference in the AR as measured via the two methods, AR-Sb and AR-mPCR, are indicated. Molecular data and case numbers for 30 PM female participants are presented in Table S1. Marker = 1 Kb DNA size ladder marker; C1, normal female, negative control, and C2, full mutation male, positive control. Supporting data are provided in Table S1.

**Figure 2:**
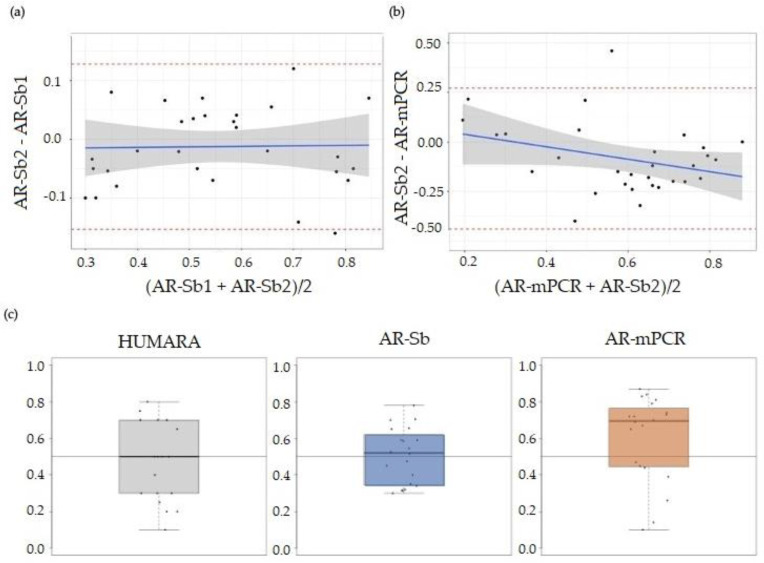
(**a**) Bland–Altman plot of AR-Sb1 and AR-Sb2 (repeated Southern blot measurements). The red dashed lines indicate two standard deviations from the mean difference. The blue line shows a linear regression fit of the difference between measurements via the mean of the measurements. (**b**) Bland–Altman plot of AR-Sb and AR-mPCR. The red dashed lines indicate two standard deviations from the mean difference. The blue line shows the linear regression fit of the difference between methods via the mean of the methods. (**c**) Boxplot of XCI and AR using Southern blot or mPCR for all individuals with an informative HUMARA assay (n = 20). Heavy lines on each box show the group median, lower and upper box edges show the 25th and 75th percentiles, respectively, and lower and upper “whiskers” show the smallest and largest observations, respectively, that lie within 1.5 interquartile ranges (IQR) of the box edges. Observations lying more than 1.5 IQR from the box edges, if any, are shown as points.

The AR for all 30 participants was determined using both the AR-mPCR and AR-Sb assays. As seen in Figure 1a, the AR-mPCR values for this patient cohort were generally higher than those obtained via AR-Sb. Figure 1b shows the Sb data for a subset of the patient cohort. There was a statistically significant correlation (*p* < 0.001) between AR-Sb1 and AR-Sb2 (repeated Southern blot measurements) (Pearson correlation of 0.919 (0.84, 0.96); Figure 2a). In contrast, a comparison of AR-Sb and AR-mPCR showed a Pearson correlation (95% Cl) of 0.637 (0.36, 0.81) with *p* < 0.001 and the confidence intervals for the two correlation coefficients that do not overlap (Figure 2b). The downward slope of the regression line suggests that the difference between the methods is larger for larger AR values.

However, as shown in Figure 2c, the HUMARA experiment revealed an approximately normal distribution of alleles with a median of 0.50, supporting the notion that there is little or no bias in allele distribution in this population. The AR-Sb assay shows an allele distribution with a median AR of 0.52. This is consistent with a previous report comparing the results obtained from the HUMARA assay and Sb in 100 women with the PM [44], suggesting reasonable concordance between the assays. In contrast, the AR-mPCR assay showed a median AR of 0.70. While the difference in the AR distribution did not rise to the level of statistical significance when the entire data set was compared, when the samples with the top 50% of AR-mPCR values were compared, the difference between the AR-Sb and AR-mPCR results was significant at *p* < 0.05 (Mann-Witney U test; *p* = 0.00116), whilst the bottom 50% were not significantly different (*p* = 0.65). This lends weight to the hypothesis that the mPCR assay tends to overestimate the AR in our patient cohort for samples in the higher AR range. The mPCR assay measures the difference in the yield of PCR product from the normal allele with and without HpaII digestion. Given the difficulty in the amplification of CGG-repeats, a situation that is exacerbated when the repeats are embedded in a long GC-rich DNA fragment, it is possible that the systematically higher AR as measured via the AR-mPCR assay reflects the improved efficiency of PCR amplification of PM alleles after HpaII digestion. Without HpaII digestion, the normal allele would be preferentially amplified, whilst, after HpaII digestion, this bias would be reduced. This would result in an apparent reduction in the fraction of normal alleles on the inactive X and thus a higher apparent AR.

#### 3.2.2. Correlation between AR-Sb or AR-mPCR and Molecular Measures

Linear regression analysis of FMR1 mRNA expression level by the number of CGG repeats and either AR-Sb or AR-mPCR showed that PM females with more CGG repeats had higher FMR1 mRNA levels after adjusting for AR-Sb (*p* = 0.03) or after adjusting for AR-mPCR (*p* = 0.05). As expected, participants with higher AR-Sb or higher AR-mPCR showed significantly lower FMR1 mRNA after adjusting for CGG repeats; *p* = 0.03 and *p* = 0.04, respectively. The ANOVA revealed that the length of the CGG was not significantly related to the number of AGG interruptions (F test *p* = 0.86).

**Figure 3:**
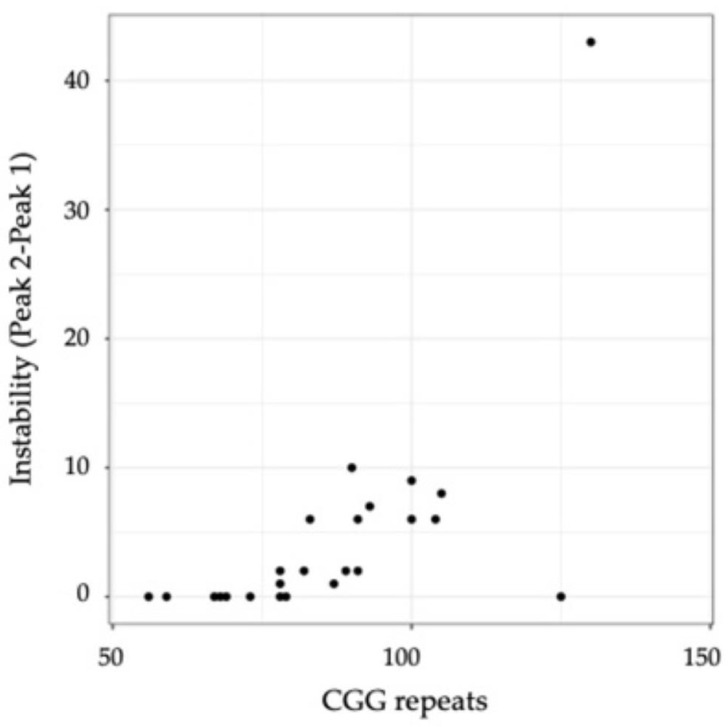
Scatterplot of instability (measured as peak 2–peak 1) as a function of the number of CGG repeats. The statistical details are shown in Table S2.

Linear regression analyses of AR-Sb and AR-mPCR showed no significant association with the number of CGG repeats (*p* = 0.12 and 0.52, respectively) or instability (*p* = 0.189 and 0.615, respectively). However, instability was significantly higher for PM participants with greater numbers of CGG repeats (*p* < 0.001; Figure 3), but no correlation was seen between instability and the number of AGG interruptions (ANOVA F test *p* = 0.0914). Furthermore, the results of the linear regression analysis revealed that instability was not significantly associated with the level of FMR1 mRNA (*p* = 0.145).

#### 3.2.3. Correlation between AR-Sb or AR-mPCR, FMR1 Molecular Measures, and Clinical Data

FXTAS was seen in 40% of PM carriers and FXPOI in 46% of carriers. The frequency of FXTAS was inversely correlated with AR-mPCR values (*p* = 0.03), while the frequency of FXPOI did not correlate with AR-mPCR. Moreover, neither FXTAS nor FXPOI frequency correlated significantly with AR-Sb (*p* = 0.11 and 0.07, respectively). A lack of correlation between FXPOI and AR is consistent with previous reports using the HUMARA assay and Southern blotting approaches [44,45,46].

As expected, a significant correlation was observed between CGG repeat number and *FMR1* mRNA expression levels, confirming previous studies [47], while no significant correlation was observed between CGG repeats and AR (by either AR-Sb or AR-mPCR). Furthermore, *FMR1* mRNA expression levels did not correlate with any of the clinical outcome measures included in this study (depression, number of medical conditions, BDS-2, and IQ).

**Figure 4:**
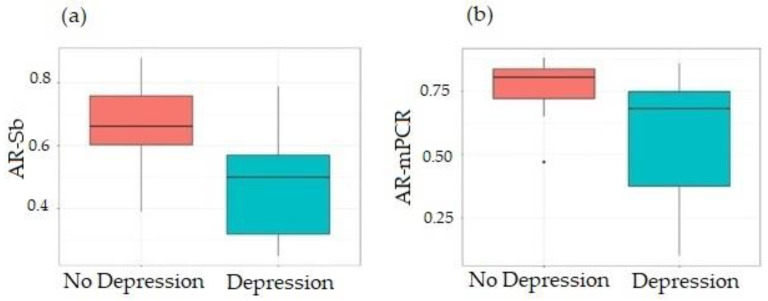
Boxplot of the relationship between AR via Southern blot (**a**) and mPCR (**b**) and a diagnosis of depression. Heavy lines on each box show the group median, lower and upper box edges show the 25th and 75th percentiles, respectively, and lower and upper “whiskers” show the smallest and largest observations, respectively, that lie within 1.5 interquartile ranges (IQR) of the box edges. Observations lying more than 1.5 IQR from the box edges, if any, are shown as points. The statistical details are provided in Table S2.

In terms of FXAND, 20 PM allele carriers (66.7%) were diagnosed with depression, with the likelihood of depression being significantly reduced for carriers of PM alleles with greater AR-Sb (*p* = 0.02, Figure 4a) or marginally significant for carriers of PM alleles with higher AR-mPCR (*p* = 0.06, 335 Figure 4b). Depression was not significantly associated with instability (*p* = 0.14). In this sub-cohort, nine (9/20, 45.0%) had a child with FXS, while 11 (11/20, 55.0%) had depression but did not have a child with FXS. Furthermore, five participants without depression had children with FXS. Logistic regression showed no significant association between the occurrence of depression and parenting a child with FXS (*p* = 0.883). The average years of education in participants with depression (data were available for 16 participants) was 15.9 ± 2.8, and 15.2 ± 1.8 years in those without depression (data were available for 5 participants). This difference was not statistically significant (*t* test = 0.63, df =10.9, *p* = 0.541).

Anxiety, an FXAND-associated condition, was present in 22 of the 30 (73.3%) participants. However, the odds of anxiety did not differ significantly by AR-Sb or AR-mPCR (*p* = 0.30 and 0.14, respectively). In addition, 10/22 (45.5%) of carriers of PM alleles diagnosed with anxiety had a child with FXS, while only one PM carrier without anxiety had a child with FXS, suggesting that there was no significant association between the occurrence of anxiety and parenting a child with FXS (*p* = 0.090). The average years of education in PM females with anxiety (data were available for 17 participants) was 15.4 ± 2.7, and 17.0 ± 1.8 years in PM carriers without anxiety (data were available for 4 participants). There was not a statistically significant difference between years of education between PM carriers with and without anxiety (*t* test = −1.41, df =6.54, *p* = 0.203).

The odds of ADHD, which were presented in 6 of the 30 (20%) PM carriers, did not differ significantly by AR-Sb and AR-mPCR (*p* = 0.32 and 0.89, respectively). None of the participants were diagnosed with ASD.

Furthermore, the entire number of medical disorders associated with FXAND, including anxiety, depression, ADHD, and ASD, was significantly lower for greater AR-Sb (*p* = 0.02) and showed a trend for higher AR-mPCR (*p* = 0.054) (Figure S1). Moreover, the logistic regression analysis demonstrated that in our sample, the overall number of medical disorders connected to FXAND was not correlated with parenting a kid with FXS (*p* = 0.205).

**Figure 5:**
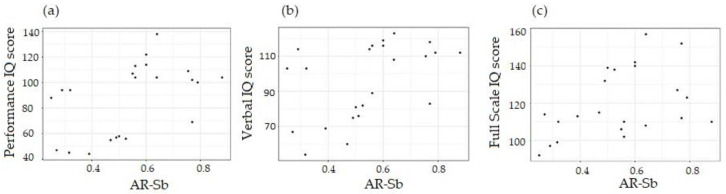
Scatterplots of performance IQ score (**a**), verbal IQ score (**b**), and FSIQ (**c**) using activation ratio determined via Southern blot. The statistical details are provided in Table S2.

Importantly, the linear regression analysis showed that carriers of PM alleles with higher AR-Sb had significantly higher PIQ and VIQ scores and FSIQ, (*p* values of 0.02, 0.02, and 0.042, respectively, as shown in Figure 5).

However, the AR-mPCR was not significantly associated with performance in any of these measures (*p* = 0.41, 0.48, and 0.13, respectively; Figure S2).

Furthermore, the results of the linear regression analysis showed that performance IQ, verbal IQ, and FSIQ were not also significantly associated with instability (*p* values of 0.85, 0.65, and 0.95; respectively, and with years of education with the Pearson correlation coefficient of 0.33 (*p* = 0.227), 0.32 (*p* = 0.229), and 0.43 (*p* = 0.107), respectively.

Finally, the results of the linear regression analysis showed that BDS-2 scores were not significantly associated with either AR-Sb or AR-mPCR (*p* = 0.12 and 0.44, respectively). BDS-2 score was not significantly associated with instability (*p* = 0.43). According to the results of the logistic regression analysis, BDS-2 scores were not significantly associated with parenting a child diagnosed with FXS (*p* = 0.387).

## 4. Discussion

It has been reported that methylation status and AR have a significant impact on the phenotype of women with *FMR1* FM [23,24]. However, the relative contributions of the AR to cognition, behavior, and other phenotypic features in female carriers of a PM allele had not been systematically studied. Here, we report our findings on the relationship between the AR, as determined using Sb and mPCR, and FXPAC and IQ.

A systematic comparison of the AR values obtained via Sb and mPCR showed a weak correlation between the AR values, with AR-mPCR values typically being higher than those found via AR-Sb. However, we observed that while higher AR-Sb was significantly correlated with higher performance IQ, verbal IQ, and FSIQ and lower odds of depression, no correlation was seen with a higher AR-mPCR for any of these conditions. Thus, our results would be consistent with the idea that the AR-Sb is a more robust metric for assessing any potential correlation with FXAND. However, the AR-mPCR did show a negative correlation with the FXTAS stage, whilst the AR-Sb did not. The reason for this is unclear and suggests that a larger study comparing the performance of the different assays is warranted.

We also showed that AR, whether measured via AR-Sb or AR-mPCR, did not significantly correlate with either CGG repeat number or with somatic instability, but that instability was significantly associated with greater numbers of CGG repeats, in accordance with previous findings [5,10,48,49,50]. In addition, this research revealed that higher ARs were associated with lower *FMR1* transcript levels for any given repeat length confirming previous findings [5,51,52].

This current study identifies the AR obtained via Sb as a relevant parameter for some fragile X-premutation-associated conditions. Specifically, our results suggest that AR-Sb would be useful in assessing the risk of specific psychiatric conditions, including depression, sleep disorders, ADHD, ASD, and anxiety symptoms, including social avoidance, interpersonal sensitivity, shyness, eye contact avoidance, social phobia, panic disorders, etc., which are common in PM carriers [2,53]. In addition, co-occurring FXAND-related conditions in carriers of PM alleles are also very common, with a higher prevalence of depression and anxiety [2,54]. Depression is more frequently reported in young carriers than in a matched control group [55], as well as in female than in male carriers of PM alleles [2,56,57], and is directly related to the number of CGG repeats [58,59]. Interestingly, our study revealed that the odds of depression were lower for female carriers with higher AR values obtained via Sb. Furthermore, it has been suggested that psychological stress and mental health disorders (i.e., depression, anxiety) are more likely to occur in mothers of children with FXS due to their own premutation status [60,61]. However, our study did not reveal an association between maternal mental health status and parenting a child with FXS.

According to some previously conducted studies examining the effects of FMR1 mutations on executive function, female PM carriers’ general cognitive abilities were unchanged [24,62]. However, others showed lower verbal IQ [63], working memory [64], numeric reasoning, and spatial-temporal processing [65], and a few studies suggested that AR values may be associated with cognitive and behavioral difficulties in PM carriers [66,67,68,69,70,71]. This current study lends weight to the significance of AR as a relevant clinical parameter and demonstrates that AR values, as measured by Sb, are associated with performance IQ, verbal IQ, and FSIQ scores. Since PM alleles are associated with lower levels of FMRP because alleles with large numbers of repeats are not translated well [26], our results are consistent with the idea that these measures are likely related to the levels of FMRP, which would be expected to show a direct relationship with the amount of the normal allele on the active X and, thus, the proportion of cells able to make normal amounts of FMRP. It also supports the idea that X inactivation contributes to clinical variability among females [72]. Recent research suggests that female PM carriers with higher education had better motor and cognitive functioning [73] and fewer executive functioning deficits [74]. However, in our study, we did not observe an association between the length of education and cognitive functioning.

In conclusion, this work demonstrates that AR is a critical moderator of PM symptoms and that using AR-Sb to test AR in individuals who carry these alleles and see whether there may be a connection to their clinical status is a beneficial method. However, we acknowledge several limitations of this study, including the small sample size, which may have had limited statistical power for the detection of significant associations. Future studies can be improved by increasing the overall sample size. In addition, it is important to note that AR in different neuronal cell types may differ from each other and from the AR in blood. Finally, future studies are warranted to investigate FMRP expression levels and the ratio AR/FMRP, in different tissues, which could further provide relevant information about the underlying molecular mechanisms leading to PM phenotypes.

## Figures and Tables

**Table 1:** Molecular measures and clinical assessment in 30 females carrying PM alleles.

Molecular Measures	Participants, n = 30			
	n	Mean ± SD	Range	Median
CGG repeat	30	89.0 ± 27.0	56–190	82.5
XCI	24	0.47 ± 0.23	0.10–0.80	0.50
AR-Sb1	30	0.56 ± 0.17	0.31–0.86	0.53
AR-Sb2	30	0.55 ± 0.17	0.25–0.88	0.55
AR-mPCR	30	0.63 ± 0.23	0.10–0.88	0.71
*FMR1* mRNA	26	2.03 ± 0.76 (St. Err)	0.07–3.97	1.95
AGG				
0	16 (53.3%) *	/	/	/
1	8 (26.7%) *	/	/	/
2	6 (20.0%) *	/	/	/
Instability	25	13.1 ± 25.7	0–43	2
**Child with FXS**	n	**%**		
	14	46.7	/	/
**FXPAC**	n	%		
FXTAS (n = 30)	12	40	/	/
FXPOI (n = 24)	11	46	/	/
FXAND (n = 30)				
Anxiety	22/30	73	/	/
Depression	20/30	67	/	/
ADHD	6/30	20	/	/
ASD	0/25	0	/	/
**Number of co-occurring conditions** **(n = 29)**	n	%		
0	3	10	/	/
1	10	34	/	/
2	10	34	/	/
3	4	14	/	/
4	2	7	/	/
**IQ scores**	mean	SD		
Verbal (n = 23)	96	22	54–123	103
Performance (n = 22)	87	28	44–138	97
Full Scale (n = 22)	120	18	92–157	113
**Years of education**	16	3	12–20	16
**Behavioral Dyscontrol Scale-2**	mean	SD		
BDS-2 (n = 22)	20.54	3.94	12–27	21.5

* Percentage of females relative to the total number, presenting with 0, 1, or 2 AGG interruptions. Abbreviations: n—number; SD—standard deviation; CGG—cytosine-guanine-guanine treplets; XCL—X chromosome inactivation; AR-Sb—activation ratio obtained using Southern blot analysis; AR-mPCR—activation ratio obtained using mPCR; AGG—adenine–guanine–guanine triplets; FXS—fragile X syndrome; FXPAC-fragile X premutation-associated conditions; FXTAS—Fragile X-associated tremor/ataxia syndrome; FXPOI—fragile X-associated primary ovarian insufficiency; FXAND—neuropsychiatric conditions recognized as fragile X-associated neuropsychiatric disorders; IQ—intelligence quotient. ADHD—attention deficit hyperactivity disorder; ASD—autism spectrum disorder. BDS-2 Behavioral Dyscontrol Scale-2. The main clinical and molecular parameters used in the analyses of this study are bolded.

## Data Availability

Data can be made available on request to the corresponding author.

## Supplementary Materials

The following supporting information can be downloaded at: https://www.mdpi.com/article/10.3390/cells12131711/s1, Figure S1: Scatterplots of a number of FXAND-related conditions using activation ratio via Southern blot (a) and PCR; Figure S2: Scatterplots of performance IQ score (a), verbal IQ score (b), and FSIQ (c) using activation ratio via PCR; Table S1: Molecular data for 30 female participants carrying PM alleles; Table S2: Supporting data for the correlation between the AR-Sb or AR-mPCR and clinical measures.
