# Supplementary material for: Activation Ratio Correlates with IQ in Female Carriers of the FMR1 Premutation
Source: Cells. 2023 Jun 24;12(13):1711. doi: 10.3390/cells12131711 (PMC10341054; doi:10.3390/cells12131711)
Supplement: Supplementary file 1 [file cells-12-01711-s001.zip › Table S1.pdf]

Table S1. Molecular data for 30 female participants carrying PM alleles.

**Cases**	**CGG**	**AGG**	**XCI**	**AR-Sb1**	**AR-Sb2**	**AR-mPCR**	***FMR1* mRNA**
**Case 1**	190	0	0.70	0.49	0.56	0.74	3.97
**Case 2**	79	1	0.30	0.58	0.51	0.45	1.81
**Case 3**	**104**	**1**	**NA**	**0.84**	**0.79**	**0.33**	**NA**
**Case 4**	78	0	0.65	0.41	0.39	0.47	2.14
**Case 5**	100	0	NA	0.4	0.32	0.28	2.19
**Case 6**	82	2	0.20	0.35	0.25	0.14	2.7
**Case 7**	67	0	NA	0.51	0.55	0.77	1.93
**Case 8**	**90**	**0**	**0.50**	**0.54**	**0.49**	**0.73**	**NA**
**Case 9**	83	0	0.50	0.37	0.316	0.1	NA
**Case 10**	67	0	0.20	0.34	0.29	0.44	2.28
**Case 11**	59	0	0.70	0.63	0.685	0.87	1.83
**Case 12**	93	2	0.25	0.78	0.639	0.84	1.93
**Case 13**	130	0	0.70	0.81	0.755	0.72	2.14
**Case 14**	78	0	0.80	0.33	0.296	0.26	2.85
**Case 15**	**91**	**2**	**0.10**	**0.37**	**0.27**	**0.67**	**2.61**
**Case 16**	**68**	**1**	**0.30**	**0.31**	**0.39**	**0.65**	**2.26**
**Case 17**	**69**	**0**	**0.5**	**0.42**	**0.486**	**0.70**	**1.7**
**Case 18**	**71**	**0**	**NA**	**0.49**	**0.525**	**0.69**	**1.85**
**Case 19**	91	0	0.70	0.66	0.64	0.69	3.23
**Case 20**	71	0	NA	0.47	0.5	0.65	1.96
**Case 21**	73	1	0.75	0.49	0.469	0.79	1.89
**Case 22**	68	1	0.30	0.64	0.76	0.83	0.77
**Case 23**	125	1	NA	0.84	0.77	0.86	1.94
**Case 24**	87	1	0.70	0.86	0.7	0.82	0.75
**Case 25**	56	0	0.70	0.57	0.6	0.72	1.79
**Case 26**	89	2	0.40	0.58	0.6	0.39	2.05
**Case 27**	100	2	0.15	0.8	0.77	0.80	NA
**Case 28**	78	1	0.5	0.49	0.56	0.79	1.88
**Case 29**	105	2	0.10	0.81	0.88	0.88	2.4
**Case 30**	127	0	0.50	0.57	0.611	0.81	0.07

Abbreviations: CGG - cytosine-guanine-guanine treplets; AGG - adenine–guanine–guanine triplets; XCL - X chromosome inactivation; AR-Sb – activation ration obtained by Southern blot analysis; AR-mPCR - activation ration obtained by mPCR.
