# Supplementary material for: Activation Ratio Correlates with IQ in Female Carriers of the FMR1 Premutation
Source: Cells. 2023 Jun 24;12(13):1711. doi: 10.3390/cells12131711 (PMC10341054; doi:10.3390/cells12131711)
Supplement: Supplementary file 1 [file cells-12-01711-s001.zip › Table S2.pdf]

Table S2. Supporting data for the correlation between the AR-Sb or AR-mPCR and clinical measures.

**Linear OR Logistic Regression Analysis of:**	**Regression Slope/ Geometric Mean Ratio OR Odds Ratio (95% CI)**	**P value**
AR-Sb by CGG repeats*	0.002 (-0.001, 0.004)**	0.1249
AR-Sb by CGG repeats*	0.001 (-0.002, 0.004)**	0.52263
FMR1 mRNA by CGG repeats and AR-Sb*	0.011 (0.001, 0.021)**<br>-0.194 (-0.363, -0.024)**	**0.0299**<br>**0.0269**
FMR1 mRNA by CGG repeats and AR-mPCR*	0.01 (0, 0.02)**<br>-0.145 (-0.283, -0.008)**	0.05371<br>0.03915
Instability by AR-Sb*	1.169 (0.921, 1.485)**	0.189
Instability by AR-mPCR*	0.954 (0.788, 1.155)**	0.615
**Instability by CGG repeats***	1.042 (1.023, 1.061)**	**< 0.001**
Instability by No of AGG interruptions*<br>1 vs. 0 AGG interruptions<br>2 vs. 0 AGG interruptions	 0.461 (0.171, 1.245)**<br>1.64 (0.577, 4.661)**	 0.120<br>0.336
FMR1 mRNA by Instability*	0.11 (-0.041, 0.26)**	0.145
Number of medical conditions by AR-Sb*	-0.063 (-0.295, 0.169)**	0.58181
Number of medical conditions by AR-mPCR*	-0.082 (-0.259, 0.094)**	0.348
**Number of FXANDs by AR-Sb***	-0.234 (-0.427, -0.04)**	**0.0197**
Number of FXANDs by AR-mPCR*	-0.151 (-0.305, 0.003)**	0.0542
**Performance IQ by AR-Sb***	7.499 (1.239, 13.76)**	**0.0213**
Performance IQ by AR-mPCR*	2.233 (-3.289, 7.754)**	0.409
Performance IQ by instability*	0.605 (-5.992, 7.203)**	0.849
**Verbal IQ by AR-Sb***	5.596 (0.766, 10.426)**	**0.0252**
Verbal IQ by AR-mPCR*	1.46 (-2.72, 5.639)**	0.476
Verbal IQ by instability*	1.09 (-3.902, 6.083)**	0.652
**Full Scale IQ by AR-Sb***	4.363 (0.174, 8.552)**	**0.042**
Full Scale IQ by AR-mPCR*	2.601 (-0.84, 6.042)**	0.131
Full Scale IQ by instability*	0.125 (-4.132, 4.382)**	0.951
BDS-2 Total Score by AR-Sb*	0.736 (-0.208, 1.679)**	0.12
BDS-2 Total Score by AR-mPCR*	0.299 (-0.484, 1.082)**	0.436
BDS-2 Total Score by instability*	-0.416 (-1.509, 0.676)**	0.433
**Depression by AR-Sb†**	0.49 (0.238, 0.84)††	**0.0224**
Depression by AR-mPCR†	0.535 (0.231, 0.89)††	0.0563

Depression by instability†	0.711 (0.426, 1.086)††	0.1374
Anxiety by AR-Sb†	0.757 (0.424, 1.257)††	0.2998
Anxiety by AR-mPCR†	0.641 (0.291, 1.04)††	0.1459
FXTAS by AR-Sb†	0.686 (0.411, 1.067)††	0.113
**FXTAS by AR-mPCR†**	0.655 (0.419, 0.935)††	**0.0331**
FXPOI by AR-Sb†	1.707 (1.014, 3.324)††	0.0677
FXPOI by AR-mPCR†	1.373 (0.934, 2.25)††	0.142
ADHD by AR-Sb†	0.761 (0.421, 1.288)††	0.324
ADHD by AR-mPCR†	0.973 (0.664, 1.515)††	0.893
Parenting a child with FXS by AR-Sb†	1.088 (0.713, 1.685)††	0.696
Parenting a child with FXS by AR-mPCR†	0.838 (0.574, 1.169)††	0.315
Parenting a child with FXS by depression†	1.125 (0.231, 5.521)††	0.883
Parenting a child with FXS by anxiety†	7.5 (0.97, 158.547)††	0.0895
Parenting a child with FXS by number of FXANDs†	1.727 (0.769, 4.358)††	0.205
Parenting a child with FXS by BDS-2 Total Score†	0.897 (0.686, 1.143)††	0.387
Years of education by AR-Sb*	0.194 (-0.466, 0.853)**	0.546
Years of education by AR-mPCR*	0.196 (-0.298, 0.691)**	0.416

† Logistic Regression Analysis;
†† Odds Ratio (95% CI);
*Linear Regression Analysis

** Regression Slope/Geometric Mean Ratio

Bold – statistically significant data;

Abbreviation: AR-Sb - Activation ratio obtained by Southern blot analysis; AR-mPCR - Activation ratio was assessed by mPCR; CGG – cytosine-guanine-guanine triplet; AGG – adenine- guanine-guanine triplet; IQ - intelligence quotient; FXAND - fragile X-associated neuropsychiatric disorders; FXTAS - fragile X-associated tremor/ataxia syndrome; FXPOI - fragile X-associated primary ovarian insufficiency; ADHD - attention deficit hyperactivity disorder; FXS - Fragile X syndrome; BDS-2 - The Behavioral Dyscontrol Scale-2.
