# Supplementary figures and images for: Activation Ratio Correlates with IQ in Female Carriers of the FMR1 Premutation
Source: Cells. 2023 Jun 24;12(13):1711. doi: 10.3390/cells12131711 (PMC10341054; doi:10.3390/cells12131711)

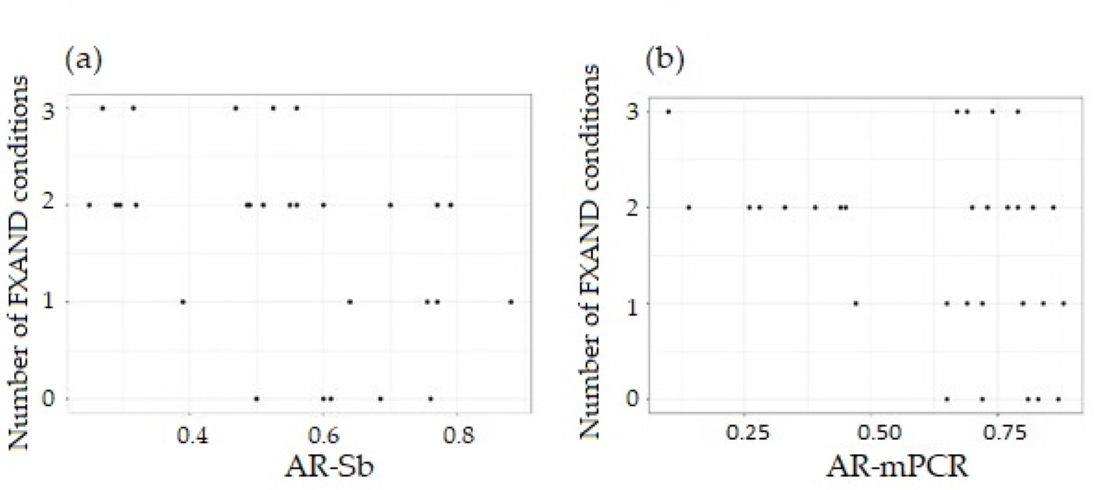

Supplement: Supplementary file 1 [file cells-12-01711-s001.zip › Figure S1 Scatterplots of a number of FXAND-related conditions using activation ratio via Southern blot (a) and PCR.jpg]

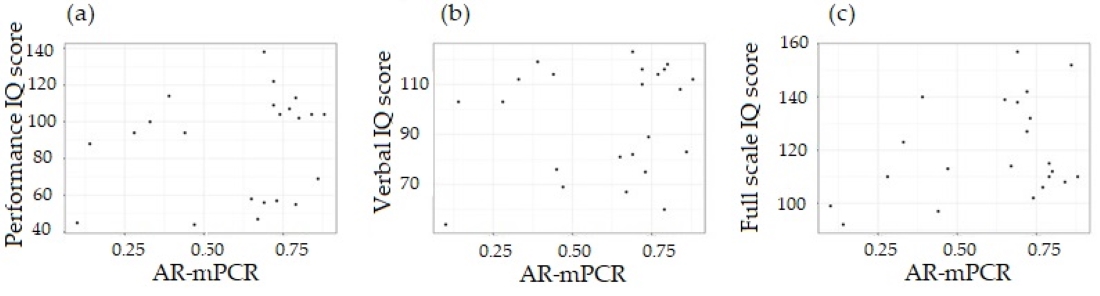

Supplement: Supplementary file 1 [file cells-12-01711-s001.zip › Figure S2 Scatterplots of performance IQ score (a), verbal IQ score (b), and FSIQ (c) using activation ratio via PCR.jpg]
